# Application of Esophageal Sponge Cytology in Screening Esophageal Squamous Cell Carcinoma in a High‐Risk Region of China

**DOI:** 10.1002/cam4.70467

**Published:** 2025-01-24

**Authors:** Shu Huang, Xuexiang Gu, Hailang Zhou, Yadong Feng, Ruihua Shi, Wei Wang, Aijun Zhou, Jie Lin

**Affiliations:** ^1^ Department of Gastroenterology Lianshui People's Hospital Affiliated to Kangda College of Nanjing Medical University Huai'an China; ^2^ Cancer Institute of Jiangsu Province, Jiangsu Cancer Hospital The Affiliated Cancer Hospital of Nanjing Medical University Nanjing China; ^3^ Department of Gastroenterology, Zhongda Hospital Southeast University Nanjing China

**Keywords:** diagnosis, esophageal high‐grade intraepithelial neoplasia, esophageal sponge cytology, esophageal squamous cell carcinoma, screening

## Abstract

**Objective:**

To investigate the feasibility and accuracy of esophageal sponge cytology in screening esophageal squamous cell carcinoma (ESCC).

**Methods:**

From May 2021 to June 2022, an opportunistic screening was performed in people aged 40–75 from a high‐risk region for ESCC. Using an esophageal cell collector that was independently developed in China for esophageal sponge cytology, a positive cytology was determined as detection of atypical squamous cells or more severe lesions. The safety, tolerability, and accuracy of esophageal sponge cytology were compared with those of pathological examination after endoscopy and biopsy.

**Results:**

A total of 1581 eligible participants were involved in the screening program, including 61 (3.86%) with high‐grade lesions pathologically confirmed by endoscopic biopsy. No serious adverse events were reported during sampling. Major adverse events included vomiting during sampling (*n* = 2, 0.13%) and sore throat after sampling (*n* = 36, 2.27%). The majority of participants showed good tolerability and accessibility (*n* = 1568, 98.80%) accessed by the visual analogue scale (VAS). Esophageal sponge cytology offered a satisfactory diagnostic accuracy in screening advanced esophageal epithelial lesions, with a sensitivity of 98.36% (95%CI: 90.02%–99.91%) and a specificity of 91.15% (95%CI: 89.97%–92.84%), and a negative predictive value up to 99. 92% (95%CI: 99.53%–99.99%).

**Conclusion:**

Esophageal sponge cytology is a highly feasible, safe, and accurate screening method for ESCC in a high‐risk region of China.

**Trial Registration:**

First Affiliated Hospital of Naval Medical University: NCT04609813

## Abstract 2

We used an esophageal cell collector that was independently developed in China for screening ESCC in high‐risk regions of China, which showed that esophageal sponge cytology is a safe, accurate, and convenient method for screening ESCC.

## 1 Introduction

The Global Cancer Statistics 2022 estimated 224,012 new cases of esophageal cancer and 187,467 related deaths in China, accounting for 43.84% and 42.13% of the global numbers, respectively. Esophageal cancer is the fifth leading cause of cancer‐related deaths in China [1]. About 90% of esophageal cancer patients are diagnosed with esophageal squamous cell carcinoma (ESCC), a dominant histological subtype [2, 3].

ESCC has a prognosis closely related to its stage. The 5‐year overall survival (OS) of advanced ESCC is strikingly lower than 20% [4, 5]. Therefore, early screening is essential to enhance the survival of ESCC. Population‐based studies in China have confirmed that upper gastrointestinal endoscopy with Lugol's staining or narrow band imaging (NBI) can increase the early detection rate and decrease the mortality of ESCC [6, 7]. However, the feasibility of a large‐scale endoscopic screening in the community is greatly limited by its expensiveness and invasive procedures, as well as patients' low compliance [6, 8]. A cheap, non‐invasive, convenient screening test is urgently needed.

Esophageal sponge cytology is an emerging test for detecting ESCC with simple procedures and low medical cost. For example, cytosponge‐trefoil factor 3 (TFF3) has been validated for its acceptability, safety, and accuracy in detecting Barrett's esophagus [9, 10], and recommended by the British Society of Gastroenterology (BSG) as a routine screening test in primary care [11]. Consistently, esophageal sponge cytology can sensitively and specifically recognize esophageal high‐grade squamous dysplasia [12]. So far, the application of esophageal sponge cytology which was used to screen ESCC in Chinese population has been rarely reported, And which may still be inadequate to obtain a robust result for estimation of sensitivity [13]. So in the present prospective study, we explored the feasibility of esophageal sponge cytology in screening ESCC in Lianshui County, Jiangsu, China, where the incidence and mortality of ESCC are twice the national level.

## 2 Methods

### 2.1 Participants

From May 2021 to June 2022, participants at 40–75 years of age who had upper gastrointestinal symptoms (including abdominal pain, abdominal distension, belching, heartburn, acid regurgitation, and chest pain) and planned to receive an opportunistic screening (esophageal sponge cytology and endoscopy) in Lianshui County People's Hospital were prospectively included in this study. Excluded were those with alarm symptoms of dysphagia, poor eating, hematemesis, melena; history of endoscopy within the past year, esophageal intraepithelial neoplasia (dysplasia), esophageal cancer, esophago‐gastric varices, or esophageal stenosis; history of esophageal surgery or use of anticoagulants and antiplatelet drugs; contraindications to endoscopy and mucosal biopsy; and life expectancy < 5 years.

**FIGURE 1:**
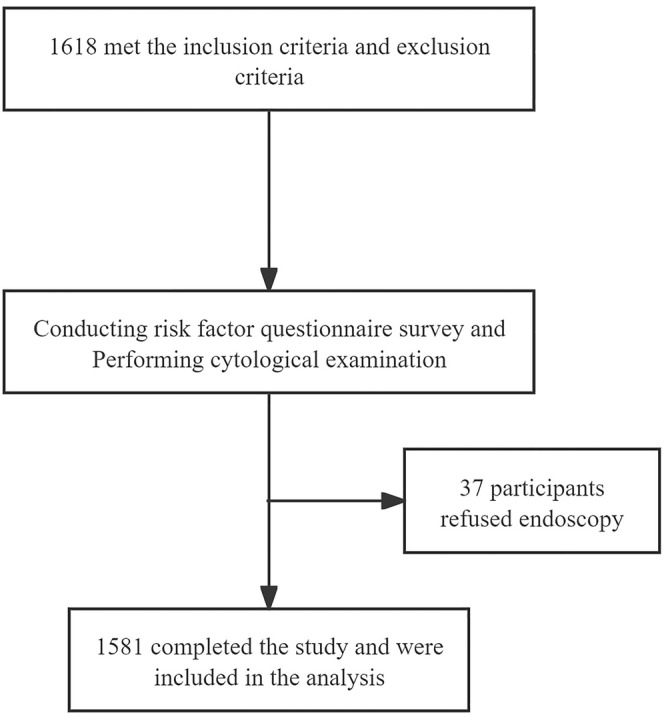
Research flowchart.

This study protocol was approved by the Ethics Committee of Lianshui County People's Hospital, the Affiliated Hospital of Kangda College, Nanjing Medical University (No. 2021226‐01). The clinical trial registration number was NCT04609813. Written informed consent was provided by each eligible participant. Demographic and baseline characteristics were recorded. The research flowchart is shown in Figure 1.

### 2.2 Procedures of Esophageal Sponge Cytology

Pre‐trained nurses in the Endoscopy Center were responsible for collecting esophageal cells using a cell collection device (Esoheal 1.0, Harbor Scientific Instrument, Hunan, China) [13]. Briefly, participants were fasted for 6 h and orally given antifoaming and mucoactive agents 30 min prior to esophageal sponge cytology sampling. They were asked to swallow a gelatine capsule in drinking 150–200 mL of 50°C water slowly, leaving approximately 20 cm of the string outside. A blue mark was labeled on the string right at the lips. Then, the encapsulated sponge contained in a dissolvable capsule was expanded into an umbrella shape slightly below the cardia. Two minutes later, the device was slowly removed by pulling the string out of the mouth, collecting cells from the esophagus. The retrieved sponges were placed into the preservative fluid (Froeasy Technology, Nanjing, China) and transferred to the Cytology Laboratory at room temperature, where cell specimens were resuspended for preparing 30 liquid‐based cytology slides. Feulgen eosin‐stained slides were finally analyzed by a digital pathology system (Froeasy Technology, Nanjing, China). The Bethesda system was adopted for reporting esophageal cytopathology which were mainly based on the degree and atypia of nuclear enlargement type and cytoplasmic maturity, including negative for intraepithelial lesion or malignant cell (NILM), squamous cell hyperplasia (SCH), atypical squamous cell (ASC), low‐grade squamous intraepithelial lesion (LSIL), high‐grade squamous intraepithelial lesion (HSIL), and squamous cell carcinoma (SCC). Based on previous study, ASC or more severe lesions (ASC^+^, including ASC, LSIL, HSIL, and SCC) were defined as positive.

During procedures of esophageal sponge cytology, sample quality and adverse events were recorded and evaluated using a structured form by pre‐trained nurses in the Endoscopy Center. Materials maintained complete inside the capsule was defined as a good quality, while a partial expansion and non‐expansion were considered as fair and poor quality, respectively. In addition, a minimum of 0.5 × 10^6^ cells per slide was qualified for diagnosis, and a second sampling was essential for participants with unqualified specimens. Subjective feelings during the sampling were evaluated using the visual analogue scale (VAS) ranging from 1 to 10 points (poor tolerability, 1–5 points; fair tolerability, 6–7 points; and good tolerability, 8–10 points). A VAS score of 6 points and higher suggested an acceptable esophageal sponge cytology.

### 2.3 Endoscopic Procedures

Endoscopy was performed after esophageal sponge cytology sampling by experienced endoscopists. NBI and 1.2% Lugol chromoendoscopy of the full‐length esophagus were conducted. Suspected tumor lesions with a diameter of 5–19 mm needed one biopsy, and larger lesions usually required multiple biopsies (two biopsies for lesions with 20–39 mm in diameter; three biopsies for lesions exceeding 40 mm in diameter). Endoscopic images were captured with a 5‐cm interval from the upper esophageal sphincter to the cardia. All participants were assessed by endoscope. Pathological examinations for biopsied specimens were performed if necessary. Histological diagnoses of esophageal biopsy specimens included normal squamous epithelium, esophagitis, squamous cell hyperplasia (SCH), low‐grade intraepithelial neoplasia (LGIN), high‐grade intraepithelial neoplasia (HGIN), and squamous cell carcinoma (SCC). Investigators were mutually blinded in performing esophageal sponge cytology and endoscopy. To unify cytology and histology diagnostic findings furthermore, low‐grade squamous intraepithelial lesion and high‐grade squamous intraepithelial lesion correspond to LGIN and HGIN, respectively.

### 2.4 Statistical Analysis

The sensitivity and specificity of the cell collection device Esoheal 1.0 were set as 0.85 and 0.8, respectively, based on previously reported data [13]. According to the statistics of annual detection rates of HGIN and ESCC in our hospital within the past 5 years, the prevalence of ESCC was set as 0.035. With an acceptable error of 0.1 and an *α* of 0.05, at least 1399 participants were required to be screened. The receiver operating characteristic (ROC) curves and Youden index were used to determine the diagnostic efficacy of esophageal sponge cytology. Statistical analyses were performed using the SPSS 26.0, R project, GraphPad Prism and MedCalc v22.009. Continuous data with a normal distribution were expressed as mean ± standard deviation (SD), and differences between groups were analyzed by the chi‐squared test or *t*‐test. *p* < 0.05 was considered as statistically significant.

## 3 Results

### 3.1 Clinical Characteristics of Participants

Between May 2021 and June 2022, an opportunistic screening for ESCC was preliminarily performed in 1618 participants. Among them, 37 participants refused endoscopy after esophageal sponge cytology sampling. Finally, 1581 eligible participants with a median age of 55 years were included, involving 842 men and 739 women.

Enrolled participants with high‐grade esophageal epithelial lesions, including HGIN and ESCC, were assigned into the case group (*n* = 61), and the remaining with normal results of endoscopic biopsy into the control group (*n* = 1520). It is found that the proportions of male participants and those with an older age were significantly higher in the case group than in the control group (both *p* = 0.000). Moreover, the proportions of participants with frequent consumptions of hot food (37.07% vs. 21.91%, *p* = 0.009) and pickled food (34.43% vs. 21.78%, *p* = 0.020), smokers (45.90% vs. 30.46%, *p* = 0.011) and those with a family history of cancers (59.02% vs. 23.49%, *p* = 0.000) were significantly higher in the case group than in the control group. Education level was significantly different between groups (*p* = 0.001) (Table 1).

**TABLE 1:** Clinical characteristics of included participants.

	Case group (*n* = 61, %)	Control group (*n* = 1520, %)	*χ* ^2^/*t*	*p*
Sex			13.372	0.000
Male	43 (70.49)	709 (46.64)		
Female	18 (29.51)	811 (53.36)		
Age (mean in year)	64.09 ± 2.42	57.39 ± 8.37	8.242	0.000
Cigarette smoking	28 (45.90)	463 (30.46)	6.531	0.011
Alcohol drinking	9 (14.75)	206 (13.55)	0.072	0.788
Hot food eating	22 (37.07)	333 (21.91)	6.751	0.009
Family history of cancer	36 (59.02)	357 (23.49)	39.635	0.000
Pickled food eating	21 (34.43)	331 (21.78)	5.422	0.020
Education level			10.665	0.001
Junior high school or above	14 (22.95)	670 (44.08)		
Primary school or below	47 (77.05)	850 (55.92)		

*Note:* Case group: 61 cases of high‐grade esophageal epithelial lesions; control group: 1520 cases with normal results through endoscopy and biopsy pathology.

### 3.2 Process of Esophageal Sponge Cytology Sampling

No serious adverse events were reported during and after esophageal sponge cytology sampling, indicating a high safety profile. Only 36 (2.27%) and 2 (0.13%) participants complained of sore throat after sampling and vomiting during sampling, respectively, which were self‐cured without further medical interventions. The majority of participants (*n* = 1562, 98.8%) had a VAS score of 6 points and above, indicating an acceptable tolerability of esophageal sponge cytology. A complete expansion of the sponge was achieved in 1523 (96.33%) participants, validating a high sampling quality. A median of 3.48 × 10^6^ (2.01 × 10^6^–4.00 × 10^6^) cells were collected during esophageal sponge cytology sampling (Table 2).

**TABLE 2:** Tolerability, safety, and sampling quality of the cell collection device.

Items	Results
Adverse events (*n*, %)	
None	1543 (97.60)
Vomiting during sampling	2 (0.13)
Throat discomfort after sampling	36 (2.27)
VAS score (*n*, %)	
8–9	1008 (63.76)
6–7	554 (35.04)
3–5	19 (1.20)
Sampling quality (*n*, %)	
Good	1523 (96.33)
Moderate	56 (3.54)
Poor	2 (0.13)
Median of scanned cells	3.48 × 10^6^

### 3.3 Cytological Versus Endoscopic and Pathological Findings

**FIGURE 2:**
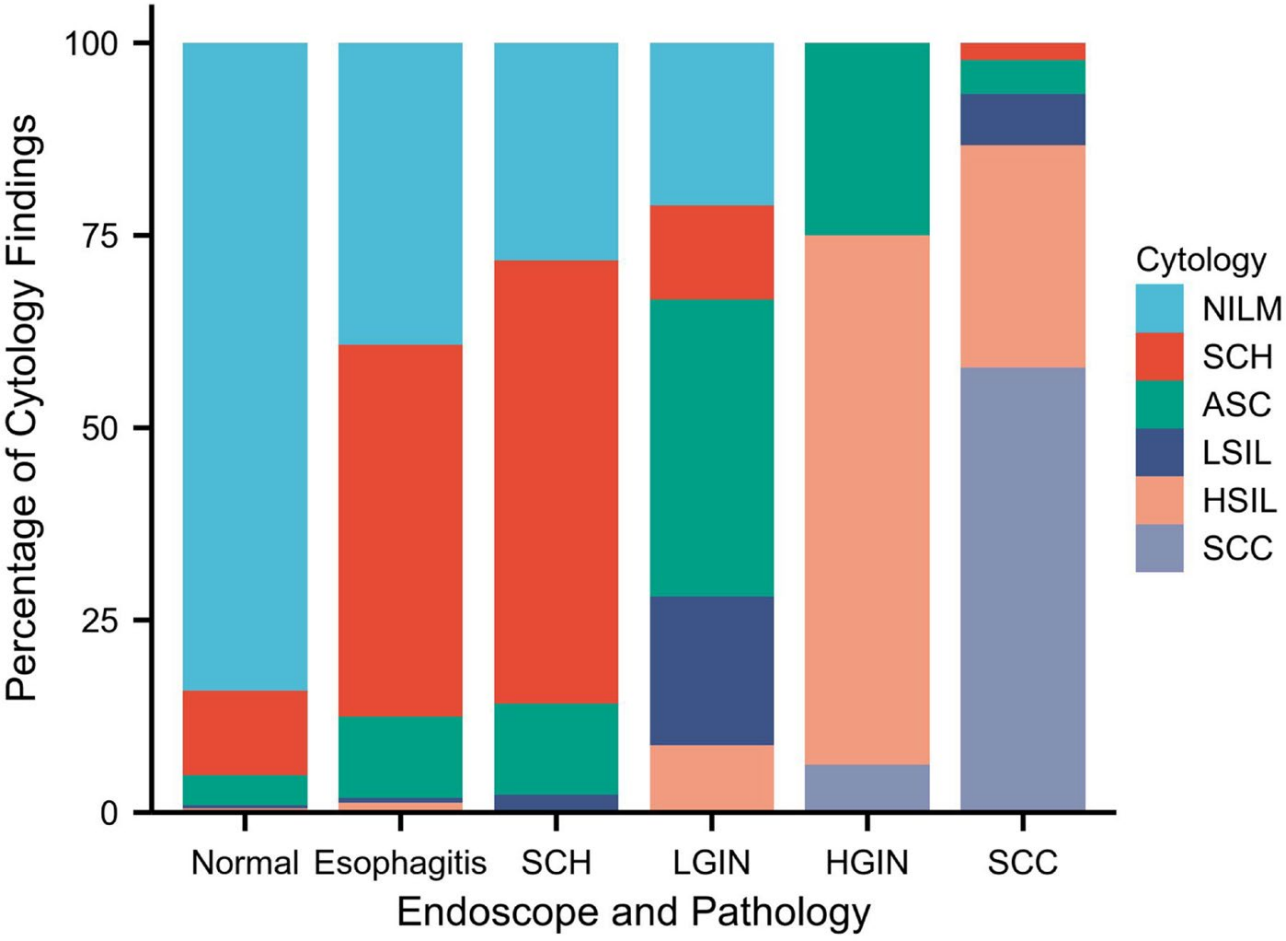
Cytological diagnosis according to pathological and endoscopic diagnosis.

Among 1581 participants were 1127 (71.28%), 265 (16.76%), 102 (6.45%), 23 (1.46%), 37 (2.34%), and 27 (1.71%) cases of NILM, SCH, ASC, LSIL, HSIL, and SCC diagnosed by esophageal spongy cytology, respectively. Pathological examinations for biopsied specimens were then performed if necessary. Endoscopy and pathology reporting showed 1225 (77.48%), 153 (9.68%), 85 (5.38%), 57 (3.60%), 16 (1.01%), and 45 (2.85%) cases of normal squamous epithelium, esophagitis, SCH, LGIN, HGIN, and SCC, respectively (Figure 2 and Table 3). Based on the comparison between esophageal sponge cytology and endoscopy and pathology, the number of endoscopies decreased significantly from 25.92 to 3.15 times, if only esophageal sponge cytology‐positive participants were transferred to an endoscopy. A misdiagnosis of ESCC was only found in 1 (1.64%) case.

**TABLE 3:** Cytological versus endoscopic and pathological findings.

Cytological/endoscopic and pathological diagnosis (*n*, %)	NILM (1127, 71.28)	SCH (265, 16.76)	ASC (102, 6.45)	LSIL (23, 1.46)	HSIL (37, 2.34)	SCC (27, 1.71)
Normal (1225, 77.48)	1031 (91.48)	134 (50.57)	48 (47.06)	6 (26.09)	6 (16.21)	0
Esophagitis (153, 9.68)	60 (5.32)	74 (27.94)	16 (15.69)	1 (4.35)	2 (5.41)	0
SCH (85, 5.38)	24 (2.13)	49 (18.49)	10 (9.8)	2 (8.69)	0	0
LGIN (57, 3.60)	12 (1.05)	7 (2.64)	22 (21.57)	11 (47.83)	5 (13.51)	0
HGIN (16, 1.01)	0	0	4 (3.92)	0	11 (29.73)	1 (3.70)
SCC (45, 2.85)	0	1 (0.38)	2 (1.96)	3 (13.04)	13 (35.14)	26 (96.30)

Abbreviations: ASC, atypical squamous cell; HGIN, high‐grade intraepithelial neoplasia; HSIL, high‐grade squamous intraepithelial lesion; LGIN, low‐grade intraepithelial neoplasia; LSIL, low‐grade squamous intraepithelial lesion; NILM, negative for intraepithelial lesion or malignant cell; SCC, squamous cell carcinoma; SCH, squamous cell hyperplasia.

### 3.4 Diagnostic Accuracy of Esophageal Sponge Cytology

**FIGURE 3:**
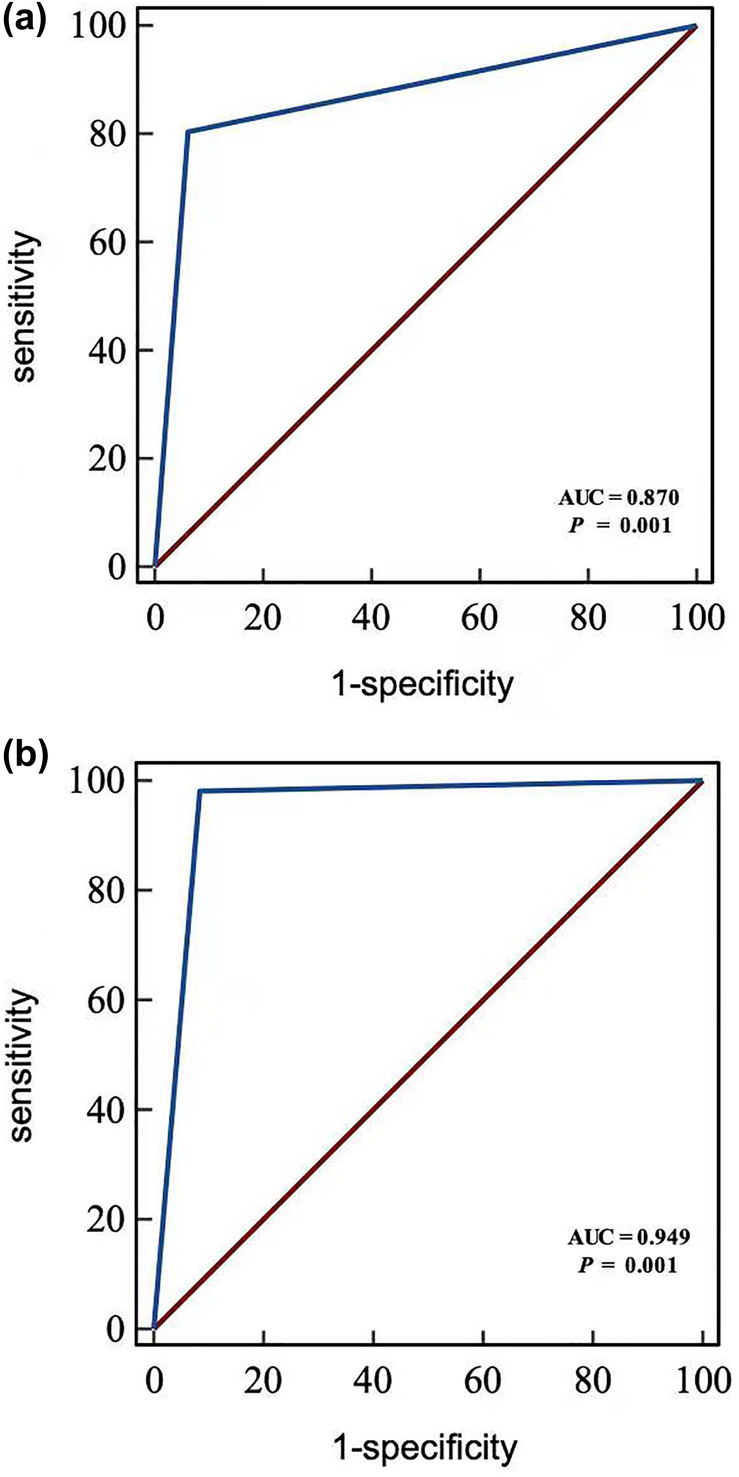
(a) ROC curves of positive esophageal sponge cytology for the diagnosis of ESCC, preinvasive carcinoma, and precancerous lesion. (b) ROC curves of positive esophageal sponge cytology for the diagnosis of esophageal epithelial advanced lesions. ROC, receiver operating characteristic; ESCC, esophageal squamous cell carcinoma.

ROC curves were plotted to assess the efficiency of positive esophageal sponge cytology in discriminating ESCC, preinvasive carcinoma (HGIN) and precancerous lesion (LGIN). The area under the curve (AUC) was 0.870 (95% CI: 0.853, 0.887), with diagnostic sensitivity, specificity, accuracy, positive predictive value, and negative predictive value of 80.51% (95% CI: 71.98%, 87.00%), 93.57% (95% CI: 92.16%, 94.75%), 92.60% (95% CI: 91.20%, 93.79%), 50.26% (95% CI: 42.94%, 57.58%), and 98.35% (95% CI: 97.49%, 98.93%), respectively. In addition, the AUC of positive esophageal sponge cytology in diagnosing advanced esophageal epithelial lesions of HGIN and ESCC was 0.949 (95% CI: 0.937, 0.960), with diagnostic sensitivity, specificity, accuracy, positive predictive value, and negative predictive value of 98.36% (95% CI: 90.02%, 99.91%), 91.15% (95% CI: 89.97%, 92.84%), 91.78% (95% CI: 90.32%, 93.04%), 31.75% (95% CI: 25.28%, 38.96%), and 99.92% (95% CI: 99.53%, 99.99%), respectively (Figure 3 and Table 4).

**TABLE 4:** Diagnostic efficacy of esophageal sponge cytology.

Positive cytology (ASC+)	ESCC, HGIN, and precancerous lesion (LGIN, HGIN, and SCC)	Esophageal epithelial advanced lesions (HGIN and SCC)
Sensitivity (%)	80.51 (71.98–87.00)	98.36 (90.02–99.91)
Specificity (%)	93.57 (92.16–94.75)	91.15 (89.97–92.84)
Accuracy (%)	92.60 (91.20–93.79)	91.78 (90.32–93.04)
PPV (%)	50.26 (42.94–57.58)	31.75 (25.28–38.96)
NPV (%)	98.35 (97.49–98.93)	99.92 (99.53–99.99)
PLR	12.53 (10.11–15.53)	11.59 (9.80–13.71)
NLR	0.21 (0.14–0.30)	0.02 (0.00–0.13)

Abbreviations: ASC+, atypical squamous cell or more severe diagnosis; ESCC, esophageal squamous cell carcinoma; NLR, negative likelihood ratio; NPV, negative predictive value; PLR, positive likelihood ratio; PPV, positive predictive value.

## 4 Discussion

Esophageal cancer was estimated as the 11th most common cancer and the seventh leading cause of cancer‐related deaths globally in 2022 [1]. In China, ESCC is the predominant histological subtype of esophageal cancer [1, 14]. Patients with early‐stage ESCC usually have a good prognosis, with a 5‐year survival of 95% [4]. However, most of ESCC cases are already in the advanced stage when they are first diagnosed, due to atypical clinical symptoms and lack of biomarkers. Advanced ESCC is a severe medical condition that poses heavy social and economic burdens [15]. Endoscopy with Lugol's staining and optical enhancement imaging of high‐risk population are common tools for screening early‐stage ESCC, although their clinical applications have been greatly restricted by invasive procedures, high cost, and low acceptability [6, 7]. Even in high‐risk regions of esophageal cancer, the relatively low prevalence weakens the cost‐effectiveness of cancer screening [6]. It is important to develop a non‐invasive, less‐expensive, and feasible screening method to identify high‐risk individuals of ESCC before endoscopy.

Balloons and brushes used to be the commonly adopted devices for esophageal cytology sampling [16, 17]. Later, sponge‐on‐a‐string test has become popular due to their collection of sufficient cells, acceptable tolerance, and high specificity and sensitivity. In recent years, esophageal sponge cytology combined with biomarkers, like TFF3 [16], tissue factor pathway inhibitor 2 (TFPI2) [18], vav guanine nucleotide exchange factor 3 (VAV3) [19], and microRNA [20], have been validated to have high efficiencies in detecting Barrett's esophagus and associated adenocarcinoma. Meanwhile, the glandular epithelial cells from the gastroesophageal junction can be collected simultaneously during sampling, so esophageal sponge cytology has been regarded to detect early gastric cardiac cancer. However, because of the sponge was put slightly below the cardia, which was unable to collect cells from other parts of the stomach, so it may not be used for non‐cardiac gastric cancer screening. Cytology tests have also showed the promising preliminary results in recognizing ESCC [12]. Esoheal 1.0, a cell collection device designed by Chinese researchers, can collect sufficient esophageal cells for screening ESCC, with high acceptability and safety. Experimental data have shown the high diagnostic sensitivity (90.0%) and the specificity (93.7%) of Esoheal 1.0 in AI‐assisted sponge cytology [13]. In the present study, no severe adverse events were reported during and after the process of esophageal sponge cytology sampling using Esoheal 1.0, consistently proving a good tolerability and a high sampling quality. Sore throat (2.27%) and vomiting (0.13%) were reported with very low incidences, all self‐cured without need for further medical interventions. A median of 3.48 × 10^6^ esophageal cells were collected using Esoheal 1.0, and then qualified for cytological interpretation. However, the number of collected esophageal cells was less than that previously reported, which could be attributed to different methods in preparing slides and staining [13].

Esophageal sponge cytology has been proved the better cost‐effectiveness as a screening method in many diseases. Benaglia et al. [21] found the cost of esophageal sponge cytology screening for Barrett's esophagus was lower compared with endoscopy ($240 vs. $299). Similarly, when it was used for screening patients with gastroesophageal reflux disease (GERD) compared with endoscopy alone, the cost of screening was reduced by 27%–29%. Therefore, we believe that it has more advantages in screening for ESCC, but more in‐depth and detailed studies are needed in the future.

The incidence and mortality of ESCC greatly vary across regions of China [22]. Lianshui County, Jiangsu Province, China is a high‐risk region of ESCC, with an incidence having increased from 47.32/100,000 in 2010 to 78.92/100,000 in 2021. To examine the safety, tolerability, and accuracy of esophageal sponge cytology in screening ESCC, participants in Lianshui County were prospectively selected from a local institution. Cancer screening is recommended to combine mass census with opportunistic screening. A study in high‐risk regions for ESCC in Jiangsu Province showed that community screening only identified 20 cases of advanced esophageal epithelial lesions among 1844 participants [13]. The detection rate was low, which may not be persuasive to demonstrate their results. So, aiming to increase the detection rate, we changed the screening methods in the study population, with an opportunistic screening. Based on strict inclusion and exclusion criteria, a total of 1581 eligible participants at 40–75 years of age were finally enrolled in this prospective study. In the end, we found 61 cases of advanced esophageal epithelial lesions among 1581 participants. We also found that the occurrence of advanced esophageal epithelial lesions was significantly correlated with age, gender, smoking, frequent consumption of pickled and hot food, education level, and family history of cancer, which are consistent with previous studies [13, 22, 23]. Hereto, we appealed for primary prevention strategies, like health education, especially in high‐risk regions of ESCC. Moreover, screening tests are particularly essential for high‐risk populations like men, the elderly, and people with a family history of cancers.

In comparison with pathological examination after endoscopy and biopsy, the diagnostic accuracy (sensitivity, specificity, and negative predictive value) of positive sponge cytology was satisfactory in detecting advanced esophageal epithelial lesions, suggesting that an endoscopy can be delayed for people with a negative sponge cytology [13]. Using the esophageal sponge cytology, only 22.92 times of endoscopy were enough to detect one case of advanced esophageal epithelial lesions, even in the high‐risk region. A previous study has shown that 99.2 times of esophagogastroduodenoscopy are needed to detect one high‐grade lesion of ESCC [13]. This discrepancy may be attributed to different screening methods in the study population (opportunistic screening vs. community screening). In addition, the number of endoscopies decreased significantly from 25.92 to 3.15 times, if only esophageal sponge cytology‐positive participants were transferred to endoscopy. Thus, esophageal sponge cytology remarkably increased the cost‐effectiveness of endoscopy. In our study, misdiagnosis of ESCC occurred in only 1 (1.64%) case who complained of violent nausea and vomiting during the process of removing the sponge, resulting in an incomplete contact between the sponge and the esophageal wall and an inadequate number of collected cells. To prevent misdiagnosis in the future, a thorough communication with participants before sampling is necessary to avoid nausea and vomiting. Besides, a gentle pulling out of the string is able to ensure a complete contact with the esophagus.

There are some limitations in our study. First, we did not analyze the relevant cost‐effectiveness in detail in our study. Second, although the required sample size was reached, this was a single‐center study, the reliability of the result would be interpreted with caution. Third, the data were finally analyzed by only one digital pathology system, which may result to the bias.

In conclusion, esophageal sponge cytology is a safe, accurate, and convenient method for screening ESCC in high‐risk regions of China, and may be replicated widely.

## Author Contributions

**Shu Huang:** methodology (lead), writing – review and editing (equal). **Xuexiang Gu:** writing – original draft (lead), writing – review and editing (equal). **Hailang Zhou:** investigation (lead). **Yadong Feng:** formal analysis (lead). **Ruihua Shi:** conceptualization (supporting), writing – review and editing (equal). **Wei Wang:** software (lead). **Aijun Zhou:** methodology (supporting), writing – review and editing (equal). **Jie Lin:** conceptualization (lead), writing – review and editing (equal).

## Ethics Statement

This study protocol was approved by the Ethics Committee of Lian shui People's Hospital Affiliated to Kang da College of Nanjing Medical University (approval number 2021226‐01).

## Conflicts of Interest

The authors declare no conflicts of interest.

## Data Availability

The data that support the findings of this study are available from the corresponding author upon reasonable request.
